# The hidden benefits of patient encounters

**DOI:** 10.1111/tct.13544

**Published:** 2022-10-05

**Authors:** Rebecca Finnegan, Orla Flanagan, Peter Cantillon, Sinead McGlacken‐Byrne

**Affiliations:** ^1^ School of Medicine NUI Galway Galway Ireland; ^2^ Discipline of Paediatrics Galway University Hospital Galway Ireland; ^3^ Discipline of General Practice NUI Galway Galway Ireland

## Abstract

**Background:**

Whilst it is widely acknowledged that health care professionals (HCPs) learn from patient encounters, research exploring what HCPs learn from their meetings with patients is relatively sparse, particularly in the context of postgraduate training. Moreover, there are few research studies that examine the contribution of patient encounters to HCP education from both HCP and patient perspectives. This study set out to explore HCPs learning from patient encounters from both HCP and patient perspectives.

**Methods:**

Qualitative descriptive design was used to conduct this study. Using purposive sampling, we recruited participants from three different groups in a single department of paediatrics in a teaching hospital. Data was collected through interviews, which were transcribed and analysed for key themes.

**Findings:**

Patients felt that they played a central role in clinical education and highlighted their ability to educate postgraduate HCPs about their lived experiences of disease. HCPs highlighted the unique insight into a chronic illness gained from patient accounts, essential to developing patient and family orientated approaches to care. HCPs reported that they developed professionally, learning to adapt their negotiation and educational strategies.

**Conclusions:**

This study highlights the importance of patient encounters as critical contributors to HCPs understanding of the lived experiences of patients with chronic disease, and offers insights into how parents view their contribution to clinical education. Much of this learning is embedded and implicit, which suggests that HCP trainees need to develop better in the moment awareness of what they are learning from their meetings with patients and their families.

## 1 INTRODUCTION

Encounters between health care professionals (HCPs) and patients are an everyday fact of life in clinical workplaces. Whilst it is taken for granted that HCPs learn from such patient encounters, there are relatively few studies that examine what HCPs are learning from their interactions with patients, particularly in postgraduate clinical education contexts.^1^ Most of the literature looking at the contribution of patient encounters to clinical learning is cited in undergraduate education.^2^ The growing awareness of what HCPs learn from their meetings with patients as undergraduates is not mirrored in the minimal research looking at postgraduate training.^3^, ^4^

In traditional clinical education, the patient was largely positioned as a passive participant: A source of interesting symptoms and signs for clinical learners and their teachers.^3^ The retreat from paternalism and the growing recognition of the importance of patient‐centred care has led to major changes in clinical education. It is now increasingly recognised that health professionals learn through “knowledge‐generating dialogues” with patients and that patients are significant contributors to the learning process.^5^, ^6^, ^7^ Patient perspectives are increasingly incorporated into health professional curricula and there is a growing use of patients as educators in undergraduate programmes.^8^ Early patient contact in undergraduate education has been shown to help students to build integrated clinical skills, increase motivation, and enhance professional identity formation.^7^, ^9^ Whilst there have been reports that recommend greater recognition of the patient's role in postgraduate education,^3^, ^4^, ^6^ research that explores this relationship between postgraduate learning and their encounters with patients in the workplace is limited. Further exploring this gap in the literature and highlighting the value of HCP‐patient encounters on learning will greatly impact the development of clinical teaching programmes at postgraduate level.

The small body of extant research looking at what postgraduate HCPs learn from patient encounters is quite disparate in its findings. For example, in a study of general practice trainers in 2017, doctors felt that they learned a lot about themselves, medical conditions, and how to live life well with an illness through their encounters with patients.^5^ In a more recent study exploring learning in the workplace, physicians reported that they often learn more from complex encounters with patients as these interactions resulted in reflecting on their practice and how they communicate with patients.^10^ In an article exploring the opportunity for learning from everyday conversations between nurses and patients, MacDonald highlights the lack of acknowledgment for the potential for learning and the deficit of literature focusing on the positive interactions between patients and experienced nurses.^11^ Furthermore, in qualitative studies exploring how nurses learn at work, there is no mention of the role of patients in this learning experience.^12^

There is perhaps a clearer consensus from patients of the value of their involvement in clinical education.^2^, ^4^, ^13^ They describe it as a positive and enjoyable experience, and some report that they felt empowered by their experience of teaching HCPs.^6^ Patients have an in‐depth knowledge of their condition and feel that their experiences benefit HCPs' learning.^4^ Indeed, physicians highlighted that they believed the patient's knowledge could potentially exceed that of some HCPs.^14^ Patients with chronic diseases are often referred to as “expert patients” as they have vast amount of knowledge about their medical condition and the health care system, which they feel should be included in medical education.^4^

Whilst the available literature offers the reader some insight into whether interactions between HCP and patients are considered a valuable learning experience, there are limited real‐life studies on this learning experience, and in particular, few studies which explore this relationship from both perspectives of these interactions. Using a small study population of general paediatric HCPs and paediatric parents/families, this study aimed to enhance understanding of the role that patient encounters play in the clinical education of HCPs and to explore the perceived value and understanding of these experiences from both perspectives.

## 2 METHODS

### 2.1 Study design

We used a qualitative descriptive design to explore the perspectives of HCPs and parents regarding the role and benefit of patient encounters on HCPs' learning. In this way, knowledge and insights were generated from the participants' personal experiences,^15^ allowing “the voices of those experiencing the phenomena of interest” to be explored.^16^

### 2.2 Setting and participants

This study was conducted within the paediatric department of a teaching hospital in Ireland in 2020. Participants were recruited by means of poster advertisement and volunteered to participate in the study. HCPs included paediatric doctors and nurses within the department. Given that parents are usually important advocates for their children within a paediatric setting, parents of children with chronic conditions who attend the department were invited to participate.

### 2.3 Data collection

A purposeful sampling strategy was employed to facilitate contextual representation^17^ as well as maximise the depth and richness of the data collected.^18^ HCPs with different levels of experience were invited to participate, ranging from junior doctors to consultants. The inclusion of a diverse participant group allowed this research to be explored through many different “lenses.”^19^ Semi‐structured interviews of between 30 and 45 min duration were conducted and audio‐recorded by the lead author. An interview topic guide developed for the project was iteratively adjusted in tandem with ongoing analysis.^20^

### 2.4 Data analysis

Audiorecordings were transcribed verbatim by the lead author.^21^ Thematic analysis was used to generate common key themes among participants using an inductive approach.^21^ This approach involved six phases, moving the researcher from familiarisation of the data, developing a coding frame, towards generation and refining of key themes.^21^ A large number of themes were identified initially. After further analysis, an infrastructure of major and minor themes was developed.^16^ NVivo 12 (QRS International) was used to structure the analysis. Reporting of the study results was compiled with reference to the Standards for Reporting Qualitative Research.^22^

### 2.5 Ethics, rigour, and reflexivity

Ethical approval was obtained from the hospital's Research Ethics Committee (ref. C.A.2289). Informed consent was obtained from all participants. This study design incorporated a number of approaches to ensure rigour was established, including data source triangulation to establish different perspectives, member checking to ensure individual perspectives were accurately reflected in verbatim, an audit trail, and a reflective diary. Reflexivity was central to all stages of data collection and analysis, ensuring transparency and highlighting the central role of the researcher in qualitative research.^23^ Given that the lead author is a paediatric trainee and is therefore an insider, rigorous attention to reflexivity using a reflexive diary and conversations with co‐authors ensured critical self‐reflection of the ways the researcher's insider perspectives could have been influencing the research process.^16^ Concerns about power differentials and coercion were mitigated by the fact that the lead researcher was not actively working in a clinical role in the study hospital.

## 3 FINDINGS

Eleven people participated in the study including five paediatric doctors (three senior and two junior doctors), three paediatric nurses, and three parents of children with a chronic illness (Table 1).

**TABLE 1:** Characteristics of the participants of this study

Index	Role	Length of experience working in paediatrics/child attending the service (years)
P1	Parent	5–10
P2	Parent	>10
P3	Parent	>10
H1	Doctor	<5
H2	Nurse	5–10 years
H3	Doctor	<5 years
H4	Doctor	5–10 years
H5	Doctor	>10 years
H6	Doctor	>10 years
H7	Nurse	>10 years
H8	Nurse	>10 years

The influence of patient encounters on learning and development was clear throughout the interviews. Experiences of HCPs suggested that the greatest learning potential from parents through these interactions was the insight into living with a chronic condition and its impact on your own professional development. Parents, as their child's advocate, acknowledged this role that they played and what they can bring to this learning relationship with HCPs.

### 3.1 Awareness of the value of patient‐HCP interactions as learning experiences

The parent accounts revealed a consistent awareness of what HCPs could learn from encounters with patients and their families. One parent emphasised a beneficial role in educating HCPs about chronic diseases “I think you can teach them about what it is like to have a chronic condition” (P1). Others spoke of helping HCPs to understand how particular chronic diseases uniquely affected their child “I am all the time educating HCPs about my child” (P3).

Echoing these comments, HCPs described encounters with patients and their families as “the best learning experience” (H2). “You are experiencing, witnessing someone's experience of having an illness in their family or having to care for that” (H4). However, much of this learning was embedded and implicit, “we often are unaware that we are actually learning” (H6).

HCPs described encounters with patients and their families as “the best learning experience.”

### 3.2 Gaining insight into the lived experiences of chronic conditions

Parents sought to highlight their role in contributing to HCPs learning beyond simply learning about an illness, to developing a more nuanced understanding of the illness experience of the child and his/her family. “I think we bring a different perspective on it, and hopefully, you get to see that” (P1) “I know my child better than anyone” (P2).

HCPs indicated that they were sometimes unaware of surrounding factors in the family and it “teaches you that there can be so much more going on” (H7). In particular, the impact that a chronic condition has on the entire family and siblings could often be forgotten. This was also voiced during the interviews with parents “They are not just these children in isolation; they are part of a family who has to care for them” (P2).

An essential finding in this study was how HCPs learn through the eyes, narratives and experiences of their patients. By virtue of their relationships with patients and their families, HCPs felt they were uniquely positioned with the opportunity “to see the whole world they live in” (H5). During patient encounters, HCPs were “stepping into their lives and get a glimpse of their reality” (H7). This insight into a chronic condition was described as the “biggest part parents bring to it” (H1). HCPs reported that given the pathophysiological focus of their training, it was difficult to truly understand the impact of a chronic illness and to appreciate the challenges that families may face. There was a consensus between parent and HCP accounts that parents and patients become “experts” about their own illnesses. Parents know their child better than anyone; they can identify the “particular patterns or idiosyncrasies of their child” (H4). From living with the condition, patients and families felt that they can teach HCPs about the day‐to‐day practicalities of living with a chronic condition. The phrase “you can't read it in a book” (H4), which commonly featured throughout the interviews, emphasised this key learning tool from patients and families.

“You cannot read it in a book.”

### 3.3 Influencing professional values and perspectives

Interviews with HCPs emphasised the influence of patient interactions on HCPs' own professional development in terms of communication skills, teaching styles, and professional values. Through interactions with patients and families, HCPs learned to adapt their communication approaches towards an increasing appreciation of patient and family needs. For example, one HCP commented about how they learned from challenging encounters with patients and their families: “You learn so much from them type of encounters, sometimes ‘difficult’ families. I think it makes you a better communicator” (H5). HCPs believe that participation in complex patient encounters helps them to adapt how they communicate and negotiate when stretched beyond the normal comfort zone of harmonious doctor‐patient communication.

Similarly, HCPs developed their set of teaching styles and strategies through the success and outcomes of patient encounters. Looking at how a patient or parent engaged with the information told to them and examining whether they understood the information were very powerful indicators of the effectiveness of a particular teaching style. Indeed, HCPs often had particular set teaching styles or strategies that they used during an encounter with a family; however, they reported that patient encounters highlighted how individual each patient or family is, and often strategies need to be adapted depending on the family in front of you. Therefore, with each encounter, HCPs developed new and improved versions of their teaching styles. “You go in with an idea and it's like, whip, no we are not doing that today, this is what is going to happen today” (H7).

Patient encounters were also positioned by HCPs as influential in developing and changing professional values and perspectives. By gaining an insight into the world of the family living with a chronic condition, HCPs figured that they gained a better understanding of their lives and their goals in life. This, in turn, developed and changed their outlooks and values when it came to forming management plans, and even affected their overall professional viewpoint. “You may not be learning a fact but it changes your perspective. It changes your outlook on how families deal with these things” (H4).

## 4 DISCUSSION

Our study results indicate that patient encounters are critical contributors to HCPs understanding of the lived experiences of patients with chronic disease. Patients and parents themselves have a central role in teaching HCPs and in postgraduate educational programmes.

Patient encounters are critical contributors to HCPs understanding of the lived experiences of patients with chronic disease.

This study adds to the literature by highlighting the unique insights that HCPs gain from their encounters into the lived experiences of children and their families. Much of the learning is embedded and implicit in these encounters, which suggests the potential if the learning could be rendered more explicit.

Insights into the lived experience of families facilitated the psychosocial contextualisation of pathophysiological conceptions of disease that give HCPs a far deeper understanding of the challenges posed by chronic conditions.^24^ It was interesting to note that parent participants in this research observed that they understood how difficult it was for HCPs to understand the lived reality of chronic disease without hearing the narratives of parents and their children. Thus, encounters between patients, parents, and HCPs can be characterised as meetings between experts where parents are expert in the lived experience of chronic diseases and HCPs are experts in pathophysiology and therapeutics.^4^, ^14^, ^25^ This provides supportive evidence for the development and growth of opportunistic workplace learning into clinical education and joint collaborative learning between HCPs and patients. Patient and parent stories are key in educating postgraduate HCPs on the aspects of a chronic illness, beyond the disease itself and can be utilised in teaching formats such as parent‐led sessions with postgraduate HCPs.

Patient and parent stories are key in educating postgraduate HCPs.

In addition to contextualising HCPs' disease conceptions, this work also shows how clinical interactions with patients can influence their professional development, their values, and their understanding of illness experiences. This again highlights the unique insight gained through patient contact in the clinical environment, and through personal stories of a child and their families, which can be very difficult to generate from classroom‐based teaching. This study offers the reader important evidence of the benefit of engagement and interactions with patients on HCPs' learning. Parents, as patient advocates, express a desire to be involved in educational programmes, and similar to findings in the literature, they are very aware of the unique role they can play in HCP's professional development as a clinician.^2^, ^4^

Clinical interactions with patients can influence their professional development, their values, and their understanding of illness experiences.

### 4.1 Strengths and limitations

A key strength of this study is the added dimension of the parental perspective, acknowledging the small numbers involved in this study, which allows the reader to develop a greater understanding of the learning potential from patient encounters and the two‐way learning process.^13^, ^26^

This study focuses its attention on learning by HCPs during these encounters. Patient learning was not explored in this study. Moreover, the two professional groups included in this study are not representative of all health professions. There is a need therefore for further research that looks at the educational potential of health professional–patient encounters from patient and allied health professional perspectives. The inclusion of an outsider co‐author (a general practitioner) and a paediatrician working in another setting allows interpretation of findings from different perspectives, and a greater understanding of the findings generated in this study.

### 4.2 Implications of this study

It is clear the powerful role of patient stories and the value of integrating this teaching element into local educational programmes. Patients and parents willingness and engagement with educating HCPs is very encouraging.

Remodelling postgraduate programmes by increasing the level of patient engagement in the curriculum, utilising informal patient encounters as educational events, as well as encouraging reflection on these encounters, and incorporating parents and patients in curricula development are essential progressive developments required by educators to integrate this learning tool that is readily available.

Incorporating parents and patients in curricula development are essential progressive developments.

## 5 CONCLUSIONS

Patient involvement is integrated to a certain level in postgraduate clinical training programmes; however, the awareness and utilisation of their powerful impact on learning and the impact of patient stories on life‐long learning may not always be perceived by HCP and curricula developers. It is hoped that by increasing awareness, this valuable learning tool will be utilised throughout HCP's daily encounters with patients and incorporated into future postgraduate HCPs' curricula.

It is hoped that by increasing awareness, this valuable learning tool will be utilised throughout HCP's daily encounters with patients.

## CONFLICT OF INTEREST

The authors have no conflict of interest to disclose.

## ETHICS STATEMENT

Ethical approval was obtained from the hospital's Research Ethics Committee (ref.C.A.2289).
